# Supplementary material for: A Humanized Yeast Phenomic Model of Deoxycytidine Kinase to Predict Genetic Buffering of Nucleoside Analog Cytotoxicity
Source: Genes (Basel). 2019 Sep 30;10(10):770. doi: 10.3390/genes10100770 (PMC6826991; doi:10.3390/genes10100770)
Supplement: Supplementary file 1 [file genes-10-00770-s001.zip › Additional_Files/Additional_File7_GOTermHeatmaps.zip!/Additional_File7_GOTermHeatmaps/Golgi_membrane.pdf]

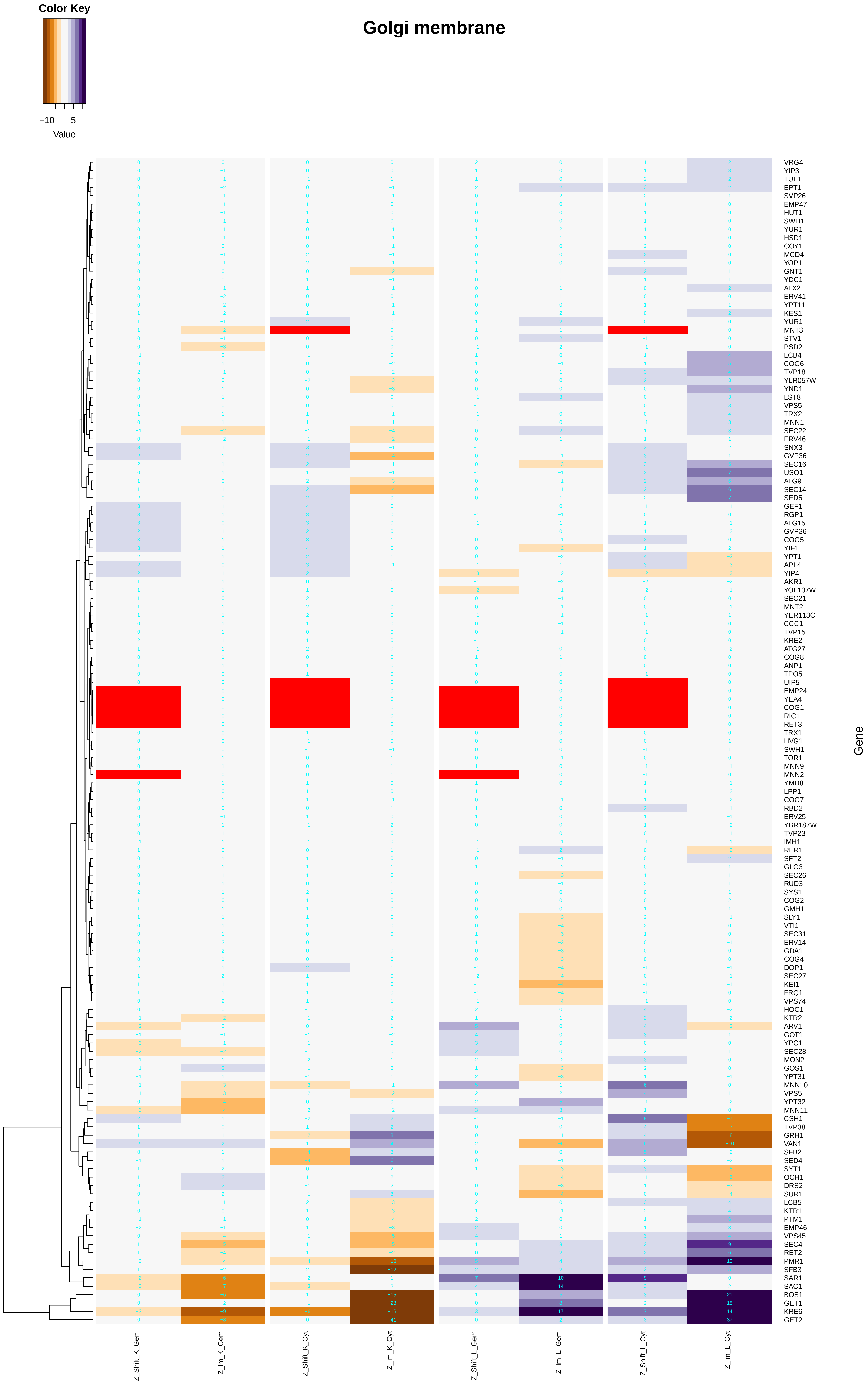

Color Key
−10
5
Value
Golgi membrane
VRG4
YIP3
TUL1
EPT1
SVP26
EMP47
HUT1
SWH1
YUR1
HSD1
COY1
MCD4
YOP1
GNT1
YDC1
ATX2
ERV41
YPT11
KES1
YUR1
MNT3
STV1
PSD2
LCB4
COG6
TVP18
YLR057W
YND1
LST8
VPS5
TRX2
MNN1
SEC22
ERV46
SNX3
GVP36
SEC16
USO1
ATG9
SEC14
SED5
GEF1
RGP1
ATG15
GVP36
COG5
YIF1
YPT1
APL4
YIP4
AKR1
YOL107W
SEC21
MNT2
YER113C
CCC1
TVP15
KRE2
ATG27
COG8
ANP1
TPO5
UIP5
EMP24
YEA4
COG1
RIC1
RET3
TRX1
HVG1
SWH1
TOR1
MNN9
MNN2
YMD8
LPP1
COG7
RBD2
ERV25
YBR187W
TVP23
IMH1
RER1
SFT2
GLO3
SEC26
RUD3
SYS1
COG2
GMH1
SLY1
VTI1
SEC31
ERV14
GDA1
COG4
DOP1
SEC27
KEI1
FRQ1
VPS74
HOC1
KTR2
ARV1
GOT1
YPC1
SEC28
MON2
GOS1
YPT31
MNN10
VPS5
YPT32
MNN11
CSH1
TVP38
GRH1
VAN1
SFB2
SED4
SYT1
OCH1
DRS2
SUR1
LCB5
KTR1
PTM1
EMP46
VPS45
SEC4
RET2
PMR1
SFB3
SAR1
SAC1
BOS1
GET1
KRE6
GET2
Gene
Z_Shift_K_Gem
Z_Im_K_Gem
Z_Shift_K_Cyt
Z_Im_K_Cyt
Z_Shift_L_Gem
Z_Im_L_Gem
Z_Shift_L_Cyt
Z_Im_L_Cyt
